# Supplementary figures and images for: pTSara-NatB, an improved N-terminal acetylation system for recombinant protein expression in E. coli
Source: PLoS One. 2018 Jul 11;13(7):e0198715. doi: 10.1371/journal.pone.0198715 (PMC6040700; doi:10.1371/journal.pone.0198715)

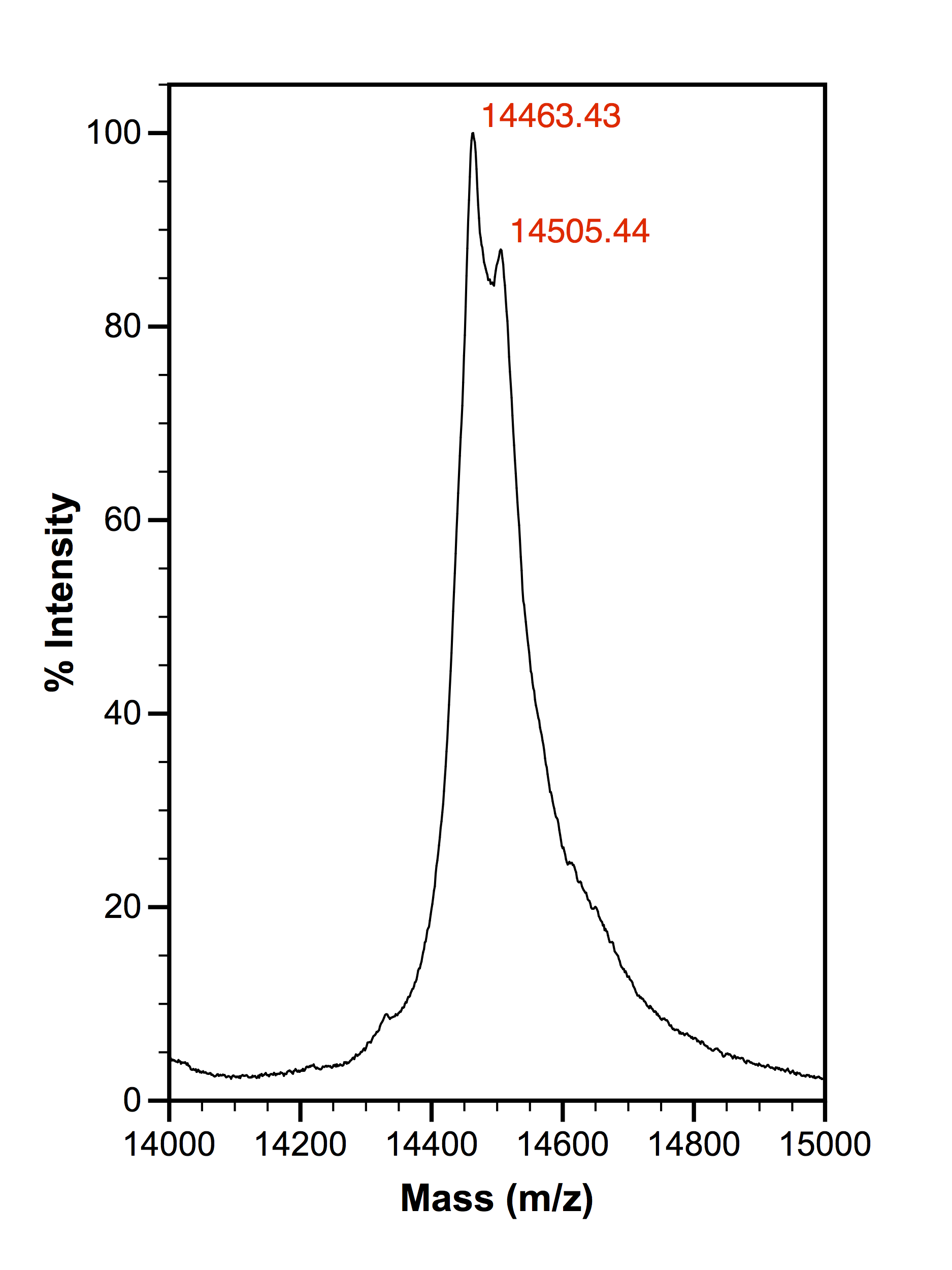

Supplement: S1 Fig — MALDI-TOF mass spectrum of αSyn purified from E. coli transformed with pET21a-alpha-synuclein+pTSara-NatB, grown in the presence of 0.2% D-glucose and induced with 1 mM IPTG (predicted MW of Nα-acetylated αSyn 14502.20 Da). (TIFF) [file pone.0198715.s001.tiff]

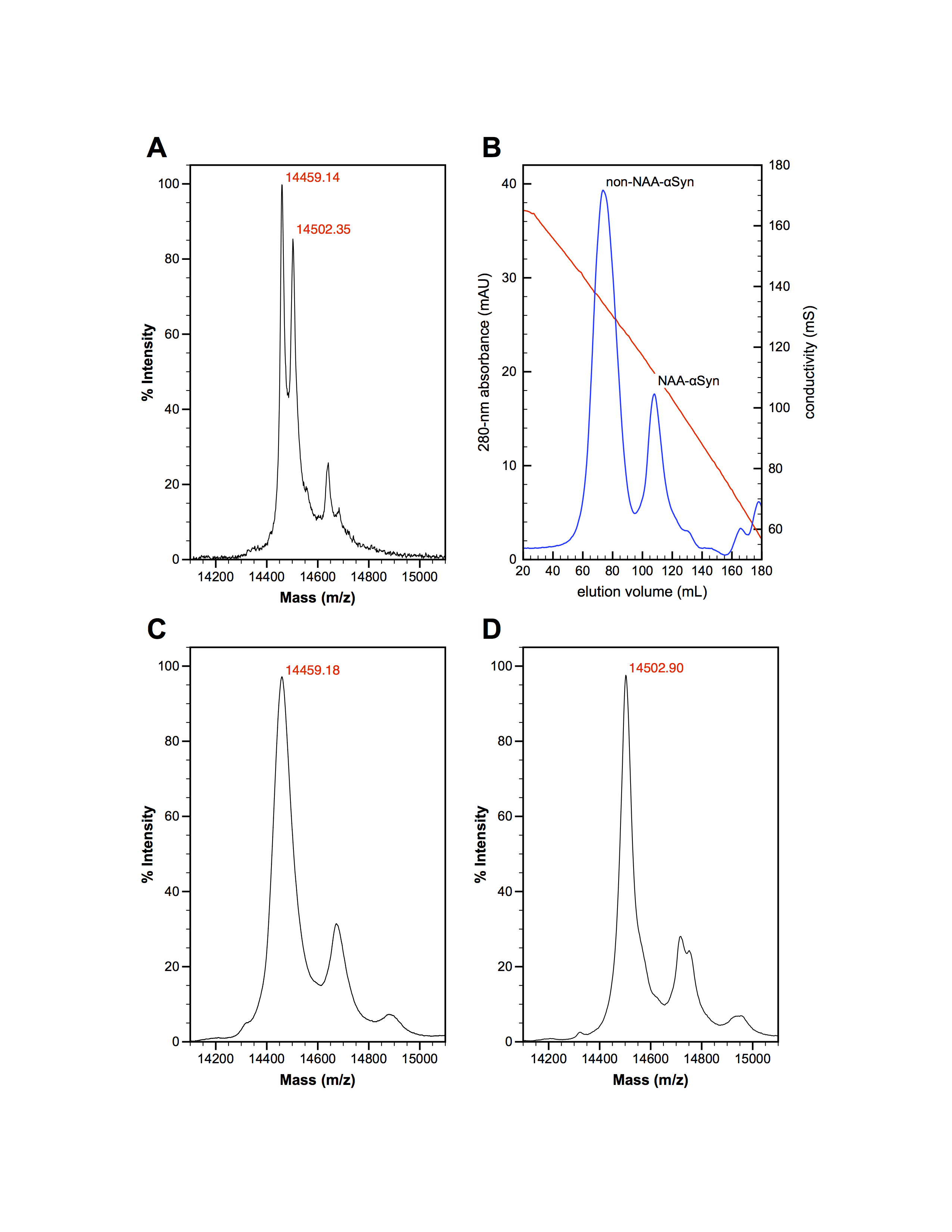

Supplement: S2 Fig — (A) MALDI-TOF mass spectrum of αSyn purified from E. coli transformed with pET21a-alpha-synuclein+pNatB and induced with 1 mM IPTG, showing a mixture of Nα-acetylated and non-Nα-acetylated αSyn. (B) Chromatogram of the HIC elution step of an aliquot from the same expression batch (in blue the 280-nm UV absorbance, in red the conductivity). HIC resolves Nα-acetylated and non-Nα-acetylated mixtures of αSyn (Nα-acetylated αSyn has a slightly higher retention volume), as confirmed by MALDI-TOF MS on the two αSyn peaks, after size-exclusion chromatography (non-Nα-acetylated αSyn, C; Nα-acetylated αSyn, D). (TIFF) [file pone.0198715.s002.tiff]

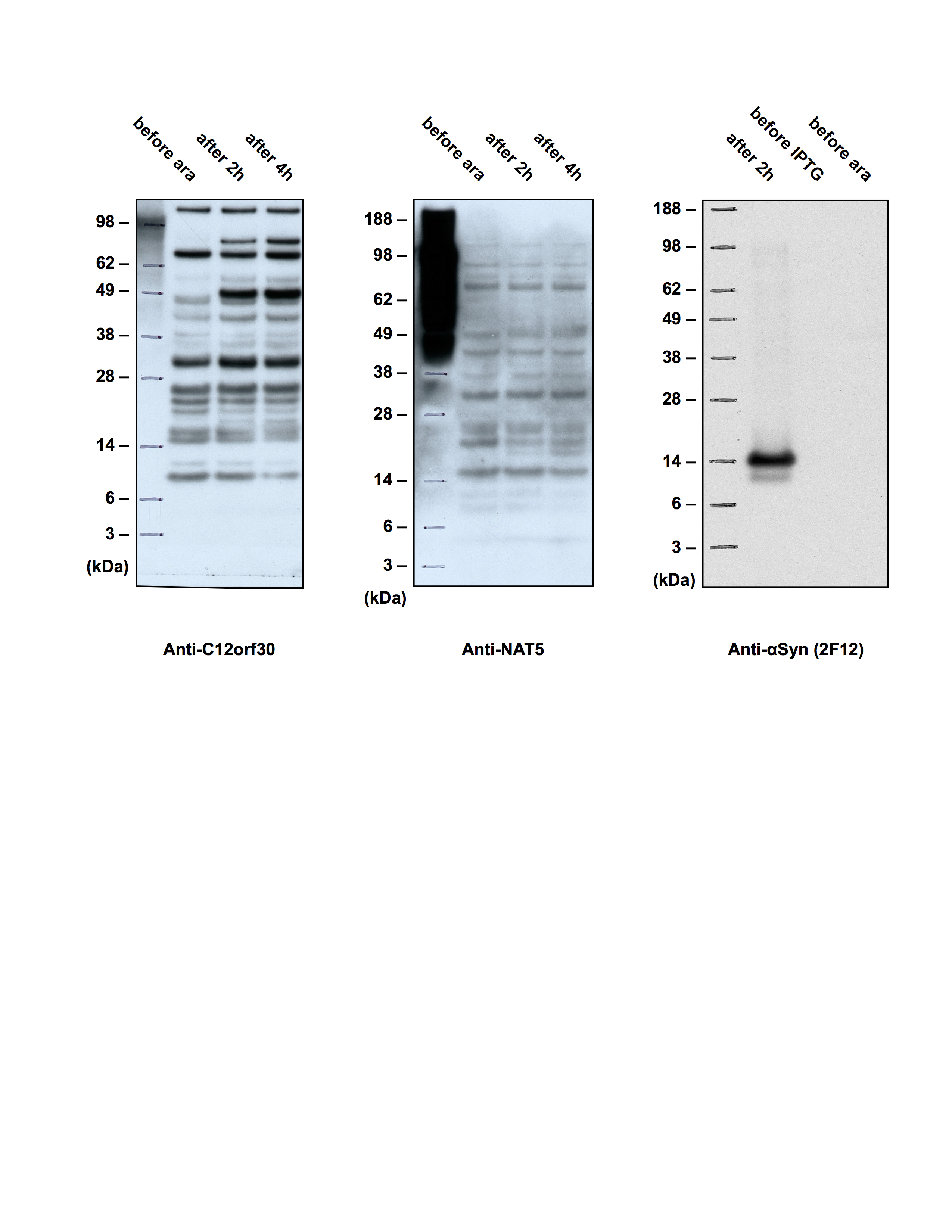

Supplement: S3 Fig — (TIFF) [file pone.0198715.s003.tiff]
